# Data-Driven Point Cloud Objects Completion

**DOI:** 10.3390/s19071514

**Published:** 2019-03-28

**Authors:** Yang Zhang, Zhen Liu, Xiang Li, Yu Zang

**Affiliations:** 1 College of Electronic Science, National University of Defense Technology, Changsha 410073, China; zhen_liu@nudt.edu.cn (Z.L.); lixiang01@vip.sina.com (X.L.); 2 School of Information Science and Technology, Xiamen University, Xiamen 361005, China; zangyu7@126.com

**Keywords:** point cloud object completion, point cloud generation, 3D reconstruction, single image, mobile laser scanning

## Abstract

With the development of the laser scanning technique, it is easier to obtain 3D large-scale scene rapidly. However, many scanned objects may suffer serious incompletion caused by the scanning angles or occlusion, which has severely impacted their future usage for the 3D perception and modeling, while traditional point cloud completion methods often fails to provide satisfactory results due to the large missing parts. In this paper, by utilising 2D single-view images to infer 3D structures, we propose a data-driven Point Cloud Completion Network (PCCNet), which is an image-guided deep-learning-based object completion framework. With the input of incomplete point clouds and the corresponding scanned image, the network can acquire enough completion rules through an encoder-decoder architecture. Based on an attention-based 2D-3D fusion module, the network is able to integrate 2D and 3D features adaptively according to their information integrity. We also propose a projection loss as an additional supervisor to have a consistent spatial distribution from multi-view observations. To demonstrate the effectiveness, first, the proposed PCCNet is compared to recent generative networks and has shown more powerful 3D reconstruction abilities. Then, PCCNet is compared to a recent point cloud completion methods, which has demonstrate that the proposed PCCNet is able to provide satisfied completion results for objects with large missing parts.

## 1. Introduction

**Figure 1:**
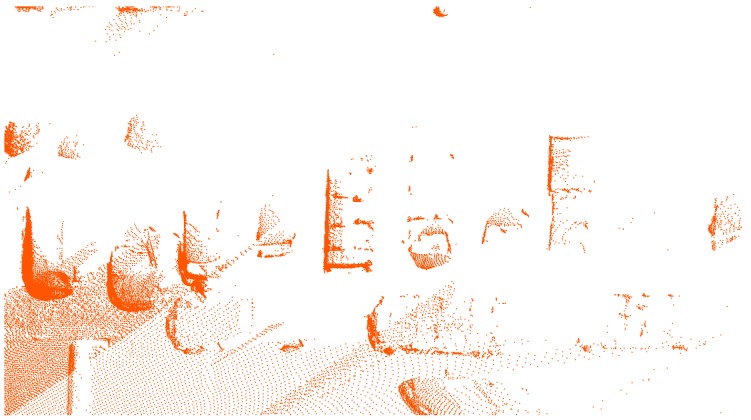
The scanned point clouds of a parking place.

As one of the most important devices to obtain 3D point clouds, laser scanning technique has developed rapidly. Scanned data have been widely used in various areas in recent decades, such as in automatic driving [1], high precision maps [2], virtual reality (VR), augmented reality (AR) [3,4], etc. However, limited by the scanning conditions, the scanned objects are often seriously incomplete. Various factors may influence LiDAR point densities and spatial distributions, for example, Balsa-Barreiro et al. [5,6] analyse variations in point density across different land covers with an airborne oscillating mirror laser scanner. Figure 1 shows an example of a parking place (acquired by the mobile scanning system RIEGL VMX-450), where most of the cars are incomplete due to the occlusion. These is a common yet challenging problem in completion for the point cloud objects.

Previous completion methods usually focus on filling in small parts, where the basic structure is relatively complete. A. Ley et al. [7] propose a simple convex optimization formulation that exploits geometric constraint, which has been demonstrated in denoising point clouds and filling in small holes on E-SAR data. Z. Cai et al. [8] come up with an occluded boundary detection method based on the last-echo information, but is only fit for on small-footprint LIDAR point clouds [9]. For the airborne laser scanning system, the data is affected by occlusion severely in trees. G. Zhou et al. [10,11] and J. Zhang et al. [12] use specified fusions between LiDAR and aerial imagery to extract buildings or various applications to eliminate the influence of occlusion. H. Wang et al. [13] utilize Hough Forest framework for object detection. In order to deal with the occlusion from adjacent objects, they propose the distance weighted voting. Some methods detect symmetries and utilize the priori knowledge to fill in missing parts [14,15], but these methods may fail when the data is not symmetrical. We have also noticed that photogrammetry, in addition to allowing completing LiDAR point clouds, provides more detailed information in some cases related to surface textures and colors [16,17].

However, most of these methods are based on the designed feature descriptors or rules and they are limited to the small-scale completion, while in practice, the objects often suffer serious incompletion, which leads to largely failures for traditional methods, thus calling for the learning-based frameworks. As far as we know, there is no one in remote sensing that has utilised the deep-learning-based method to complete point cloud object.

The completion of large missing parts is essentially a generation problem, and some of the recent generative methods have provided beneficial inspiration. ShapeNet [18], known as a large-scale CAD dataset, has promoted the development of 3D generative methods, which can be divided roughly into two sets of methods: voxel-based and point-cloud-based methods. J. Wu et al. [19] propose a 3D Generative Adversarial Networks (3D-GANs) to predict voxelized 3D models, and have achieved superior results compared to other unsupervised methods. H. Fan et al. [20] focus on generating point clouds from a single image and come up with the point-cloud-generative network (PSGN). They use Chamfer Distance (CD) to calculate the distance between the generated model and ground truth. X. Yan et al. [21] utilize projection maps to obtain 3D spatial distribution. M. Tatarchenko et al. [22] propose the octree generating network (OGN), which has achieved state-of-the-art results among the voxel-based methods. An exception is the recent work of C.-H. Lin et al. [23]. The method produces dense multi-view projected point clouds, rather than the spatial 3D models directly.

Considering the irregular and unordered distribution of the point cloud, it is difficult to process such data under the deep-learning frameworks. To address this problem, C. R. Qi et al. [24] propose PointNet, which is a basic work for point clouds classification and segmentation. Then, the network is further improved by their following work [25], PointNet++, which learns local features with increasing contextual scales through a proposed hierarchical architecture.

**Figure 2:**
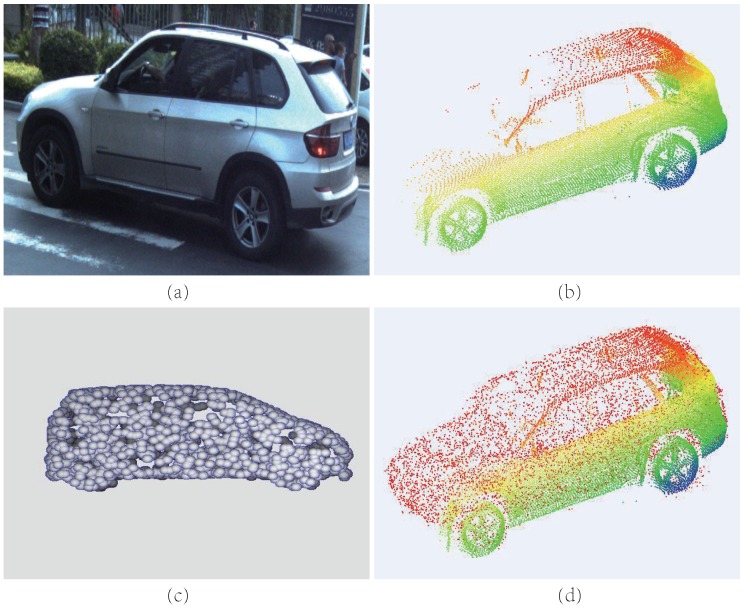
The sample images of reconstruction and completion on Mobile Laser Scanning (MLS) point clouds. (**a**) The real street images. (**b**) The scanned point clouds. (**c**) The generated point clouds (rendered). (**d**) The merged point clouds.

In order to reconstruct the object with large missing parts, inspired by the above point cloud generative networks, we propose the Point Cloud Completion Network (PCCNet), which is the first image-guided deep-learning-based scanning object completion framework by utilising 2D single-view images to generate complete point cloud models. To jointly consider the 2d and 3d information, an attention-based module is designed to fuse the 2d and 3d features adaptively, then the decoder learns to construct the whole model. Furthermore, to obtain consistent spatial distribution from multi-view observations, a projection supervision scheme is offered to provide consistent multi-view reconstruction results. Figure 2 is an overview of our method: (a) and (b) are the input of PCCNet, and (c) is the output of PCCNet (intermediate result) and (d) is the final result after aligned by the Iterative Closest Point (ICP) [26] with the scanned point clouds.

## 2. Network Architecture

In this section, we introduce the network framework, which completes 3D object models based on a real image. Our algorithm involves three steps: (1) We obtain the training and testing data (see in the supplementary material). (2) Then, taking the image and point clouds pairs as input, the network is trained to generate corresponding point clouds. (3) Finally, the generated point clouds are aligned with the initial point clouds to obtain complete 3D models.

### 2.1. Problem Statement

Our goal is to generate complete 3D point clouds through an original image. Using a large number of unordered points to compose an object, designated as $P={\left\{\left(x_{i},y_{i},z_{i}\right)\right\}}_{i=1}^{N}$, where *N* is the number of points. Here, to achieve a balance between a good presentation of 3D models and calculation burden, *N* is set as 1024. Points are sampled on the surface from CAD models in ShapeNet.

The network actually learns a mapping scheme from a 2D image and the incomplete model to its corresponding model, denoted as:

(1) $$P_{g}=G\left\{\left(I,T;\Phi \right)\right\}.$$

where Φ denotes the network parameters; *T* denotes the incomplete model; and *I* denotes the 2D image. For evaluation, a given incomplete model is connected with the image to form input pairs.

Then, the merging phase is to combine the aligned generative point clouds and initial point clouds:

(2) $$P=P_{g}^{\prime }+P_{I}.$$

where $P_{g}^{\prime }$ and $P_{I}$ denote the aligned generated point clouds and initial point clouds respectively.

### 2.2. PCCNet Architecture

**Figure 3:**
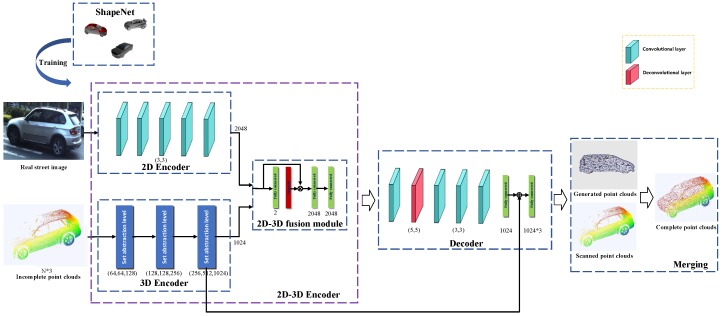
The framework of PCCNet.

To complete shapes with large holes, we propose a novel network to generate point clouds, as shown in Figure 3. Unlike conventional networks for reconstruction, our network uses two inputs: the incomplete 3D shape and its corresponding image. In the training phase, the process contains two stages to obtain the point clouds.

First, in the encoding phase, we use a 2D encoder to extract the image feature and a 3D encoder to obtain the features from incomplete shapes. Then, we design an attention-based module to fuse the 2D and 3D features, which can learn to adjust weights of the two parts adaptively. So after fusing the two features, we have an insight of the whole object, not only from the 2D form, but also from spatial and geometric information. Next, we use a decoder comprised of several convolutional and deconvolutional layers, learning to map the fused features to complete point clouds. The output is the generated point clouds as a 1024×3 matrix.

Specifically, we give a detailed illustration about the architecture. For the input, a 128×128 image and an incomplete shape with 1024 points make up the input pair, which is fed into the 2D-3D encoder. The 2D encoder contains five convolutional and ReLU layers. Then, a 2048-dimensional feature map of the image is produced. As for the 3D encoder, we adopt the basic structure of PointNet++. Three set abstraction levels, including the sampling, grouping and PointNet layers, are utilised to extract the 3D information. Thus, we obtain a 1024-dimensional feature of the 3D part. To jointly consider the 2d and 3d information, an attention-based fusion module is designed to fuse the 2d and 3d features. First, the concatenated features of the two encoders are fed into a fully connected layer and a sigmoid layer to form two weights between 0 and 1, which represent the relative significance of the two features. Then, two fully connected layers learn to further integrate them and reshape to 16×16 with 8 channels to fit for the decoder.

Inspired by the single-view generative networks, the decoder contains four convolutional layers, one deconvolutional layer and two fully connected layers, which can recover the 3D distribution from the feature space. To keep more fine-grained structures from the initial 3D models, a skipped connection from the third set abstraction level is added, such as the structure of U-Net [27]. After the last fully connected layer, the map is reshaped to a 1024×3 matrix.

### 2.3. Loss Function

Inspired by the single-view generative networks, we use the Chamfer Distance (CD) as the criterion measuring the distance between two models $S_{1},S_{2}\subseteq R^{3}$:

(3) $$d_{\mathrm{CD}}=\sum_{p\in S_{1}}\min_{q\in S_{2}}{∥p-q∥}_{2}^{2}+\sum_{p\in S_{2}}\min_{q\in S_{1}}{∥p-q∥}_{2}^{2}.$$

where $S_{1}$ and $S_{2}$ denote the generated model and ground truth; *p* and *q* denote points in these two models. CD can be conducted efficiently, and the overall distance is the mean of all points in the two shapes. Both PSGN and our experiments confirm that CD provides a good measurement of spatial distance. Additionally, we add a projection loss to train the network. At each iteration, the generated point clouds and ground truth are rotated according to the same random transformation. Then, they are projected on a 128×128 image. For every pixel, the projection pixel and its three neighbor pixels are labeled as foreground with white.

To ensure the multi-view observation consistence while capturing fine-grained parts, we adopt the the projection as an additional supervisor. Notice that there is a recent work [23] that generates multi-view projection directly and is designed for dense point cloud generation. On the contrary, PCCNet targets at real images and measure the discrepancy of projections. The projection loss is the per-pixel discrepancy between the two projected images of the generated model and ground truth:

(4) $$L_{p}=\sum_{i}{∥p_{i}-q_{i}∥}_{2}^{2}.$$

where $p_{i}$ and $q_{i}$ denote the pixels with the location *i* in the two projected images.

Experiments are carried out to compare the function of projection loss, which demonstrate the promotion in training speed and accuracy (Section 3.2). The total objective function is:

(5) $$L_{\mathrm{total}}=d_{\mathrm{CD}}+L_{p}.$$

## 3. Experiment

In this section, we provide some implementation details about the proposed PCCNet along with the employed datasets. To evaluate the capability of 3D reconstruction, first, PCCNet is compared to two single-view reconstruction methods. Then, PCCNet is compared with a state-of-the-art MLS completion approach.

### 3.1. Dataset and Implementation Details

*Dataset.* Our network is trained on ShapeNetCore55, which covers 55 common object categories with approximately 51,300 unique 3D models. To construct the image and point clouds pairs for training, we use CAD models with complex backgrounds from one fixed viewpoint (looking down at 20 degrees) to mimic the real images. Simultaneously, we sample the CAD surface to obtain point clouds. All of the sampled point clouds are normalized into a 1 m cube and centered at the origin. We split the dataset into training and testing sets in the ratio of 4:1.

In the testing phase, point clouds are generated from real street photos, together with scanning point clouds acquired by a RIEGL VMX-450 MLS system. However, at the same time it suffers from incompetent scanning especially at the back. First, we will have a brief introduction of the MLS system. Then, to have a clear view of our method, the making procedure of the training data is introduced.

**Figure 4:**
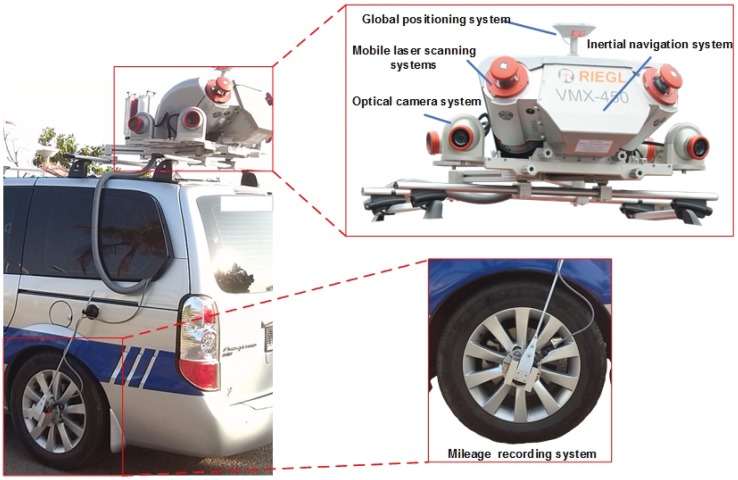
The component of our MLS system.

*MLS system.* There are mainly five parts, as shown in Figure 4, which are mobile laser scanning system, optical camera system, global positioning system, inertial navigation system and Distance Measurement Indicator (DMI). The core device is the mobile laser scanning system, i.e., a RIEGL VMX-450 MLS system, which can provide low-noise and gapless $360^{\circ }$ lines at a measurement rate of 550,000 pts/s and a scan rate of up to 200 lines/s. Meanwhile, to form the training data of picture-point-cloud pairs, the optical camera system, containing four optical digital camera to capture the surrounding environment, is taking photos at the same time. The other three systems provide assistant effects for the scanning procedure.

**Figure 5:**
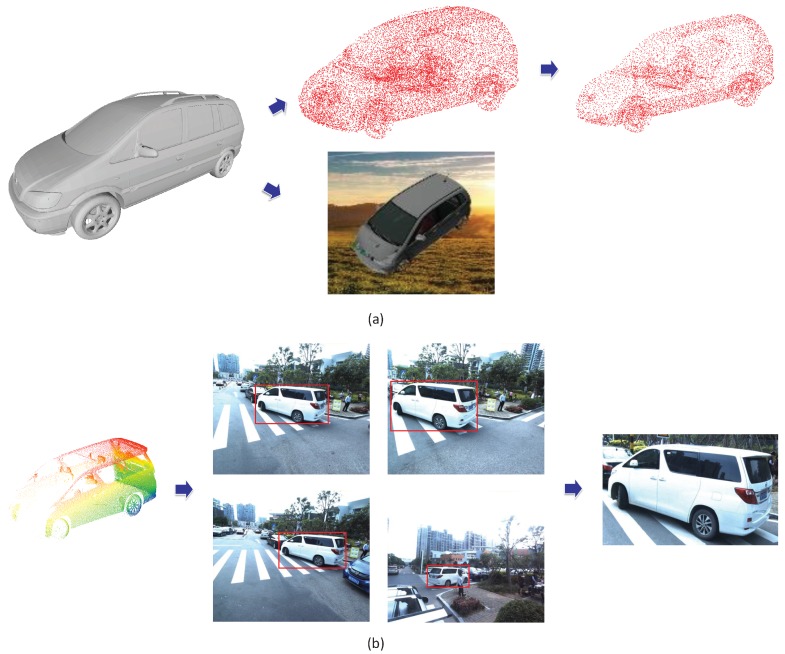
The procedure of making MLS pairs.

**Figure 6:**
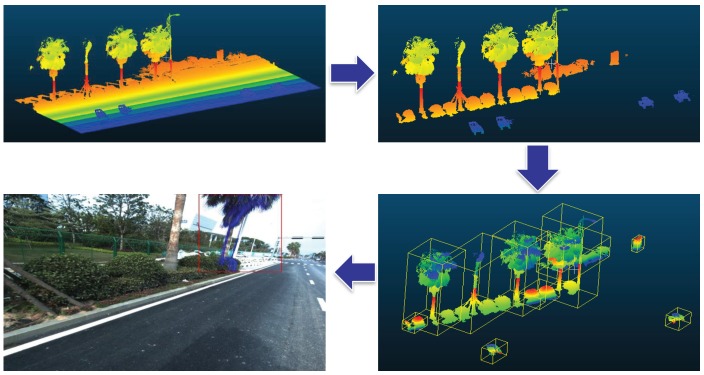
The procedure of obtaining individual MLS objects.

*The making procedure of training and testing data.* The whole data contains two parts, which are ShapeNet data for training and MLS data for testing. There are a large amount of mesh models in ShapeNet, first, we sample points on the surfaces of these meshes as the complete models. To form incomplete models, we select random planes through the center of models and cut a half. The pairing images are rendered with random selected background images. The procedure of making ShapeNet training data is shown in Figure 5a. For the MLS data, as shown in Figure 6, the first step is to remove the ground and get individual MLS objects. Then, based on the recorded parameters of each images and 3D-2D projection relationships, we are able to get accurate image and point clouds pairs. Due to the one-to-many mapping between 3D models and 2D images, with careful selection, we obtain MLS pairs for testing.

*Implementation details.* The network is programmed in the TensorFlow framework, the training optimizer uses Adam [28]. We run the code on a server with two Titan X GPUs. The network is trained from scratch with a batch size of 50 and 300 epochs in total. The learning rate automatically decays according to the setting of PointNet++. The size of the input pictures is 128×128, and the number of generated point clouds is 1024. For the 2D encoder, the kernel size of the convolutional layers is 3×3 with no padding. The parameters of the 3D encoder are derived from PointNet++: the numbers of sampled points are 512 and 128, and each local group has 64 points with the ball radius of 0.35 and 0.45. As for the decoder, the kernel size of the deconvolutional layer is 5×5. Besides, we set ReLU as the activation function.

### 3.2. Evaluation of the Proposed PCCNet

**Figure 7:**
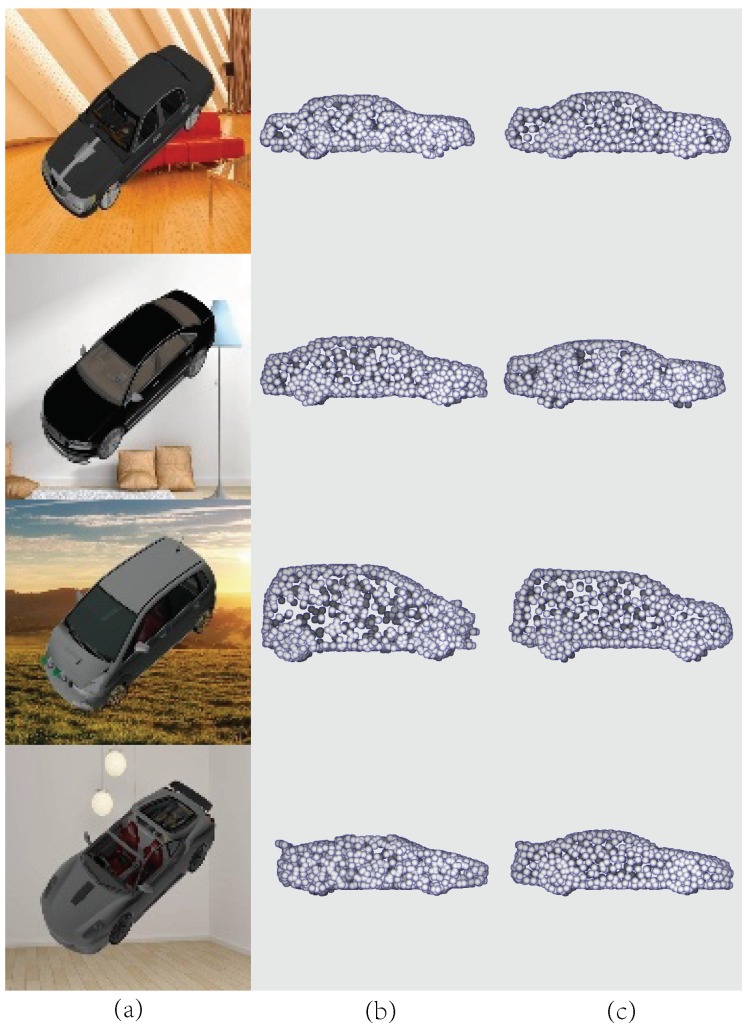
Results on rendered images. (**a**) Rendered images. (**b**) Ground truth. (**c**) Generated point clouds by PCCNet.

*Reconstruction performance of PCCNet.* To have an intuitive understanding of PCCNet, based on ShapeNetCore55, we select five categories for training and testing data. To simulate the real environment, the CAD dataset is synthesized with several real scenes. Shown in Figure 7 are four selected cars, it can be seen that the generated point clouds by PCCNet are sharing similar distribution with the ground truth.

In order to measure the attention-based fusion module, we take away the weighted branch (a fully connected layer and a sigmoid layer) and keep the two fully connected layers. The results are shown in column PCCNet_WF in Table 1 and Table 2. It can be seen that compared with the complete structure PCCNet_P, PCCNet_WF has lower accuracy.

**Figure 8:**
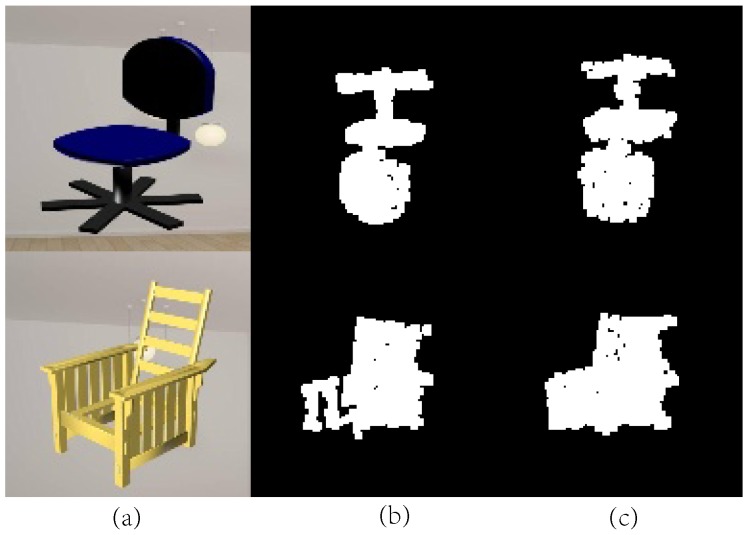
Two samples of projection from the same viewpoint. (**a**) Rendered input images. (**b**) Projection of the ground truth. (**c**) Projection of the generated shapes.

**Figure 9:**
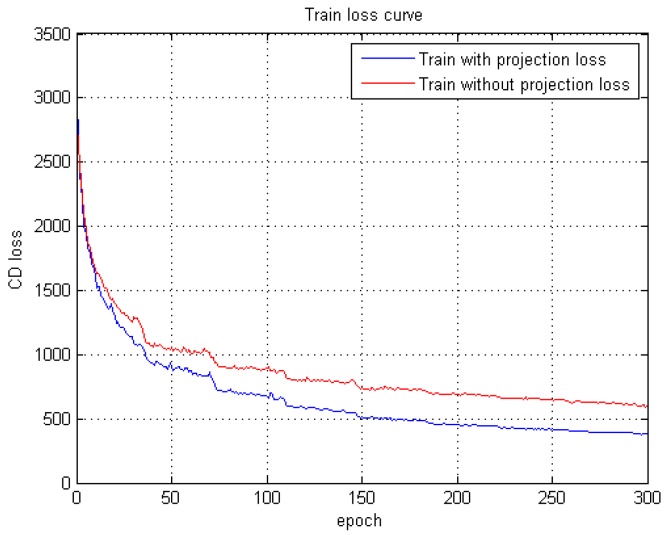
Comparison on training loss curve. The red line displays the training process without projection loss, and the blue line is with projection loss.

The function of projection is to delineate the outline of an object. Compared with volumetric methods [29] that cannot delineate some fine-grained parts, our method can exhibit more detailed parts, thus accelerating and promoting training quality. Figure 8 shows two samples of the projection results with some fine-grained parts. The training comparison is shown in Figure 9. After adding projection loss, the CD loss decreased faster, and achieved higher accuracy.

**Figure 10:**
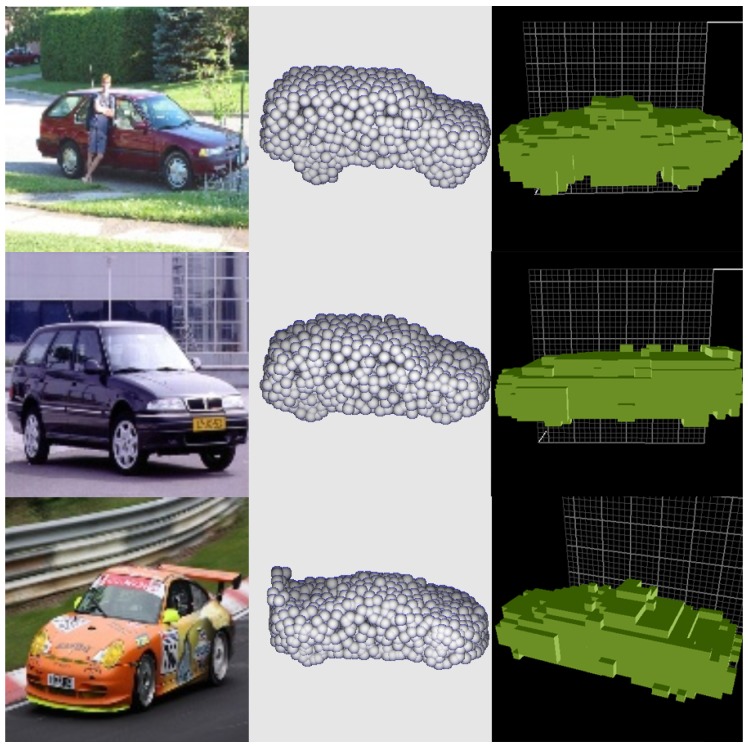
Car images from ObjectNet3D [30]. The orders from left to right: original images, the results of PCCNet and OGN.

**Figure 11:**
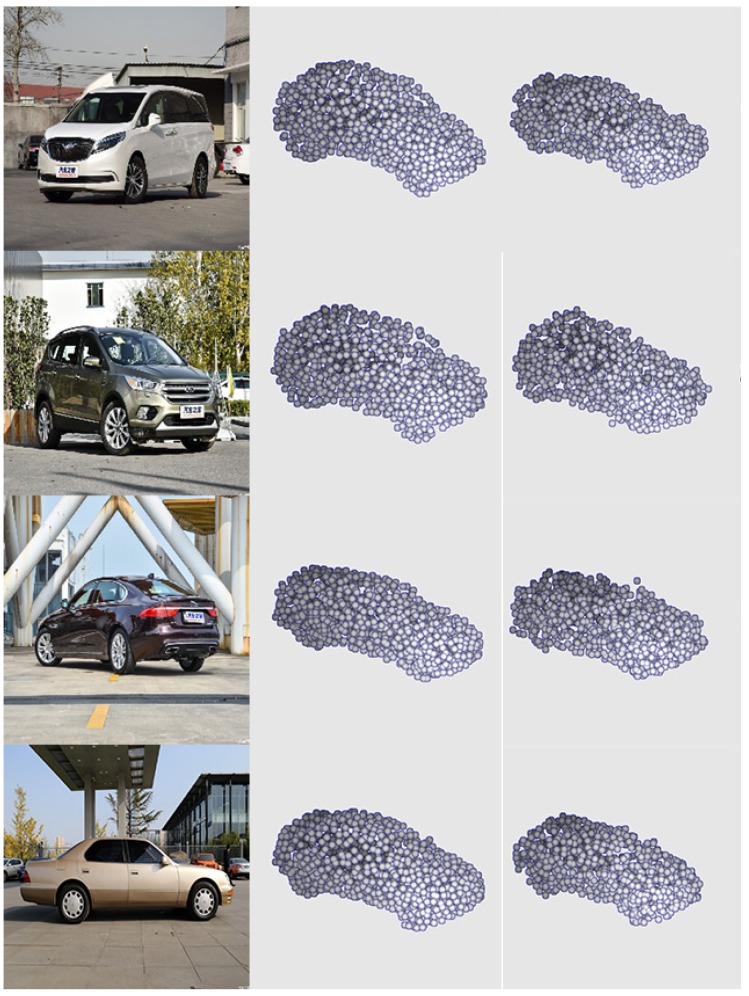
Car images from Internet. The orders from left to right: original images, the results of PCCNet and PSGN.

*Comparisons with state-of-the-art generative networks.* To evaluate the reconstruction capability, PCCNet is compared with OGN and PSGN, reported as state-of-the-art 3D object generation networks. The measurement between PCCNet and OGN is Intersection over Union (IoU), which is widely adopted by voxel-based methods. Meanwhile, the measurement between PCCNet and PSGN is CD, which is widely used by point-cloud-based methods. Comparisons with OGN and PSGN are shown in Figure 10 and Figure 11, following their original settings and displays, which demonstrate that our generated 3D models are more similar and integrated.

Statistics of the reconstruction accuracy on five categories are shown in Table 1 and Table 2. In the two tables, PCCNet_P and PCCNet_WP denote PCCNet with and without projection loss, and PCCNet_WF is without the weighted fusion module. From the results, we can see that PCCNet, PCCNet_WP and PCCNet_WF achieve the higher accuracy compared with state-of-the-art generative methods on images with complex backgrounds. Besides, PCCNet_P performs better than PCCNet_WP since the multi-view consistency is considered.

### 3.3. Comparison with Traditional Point Completion Works

Due to the scanning conditions, objects in scanned point clouds are often faced with severe incompletion. Because traditional point completion methods require roughly complete models, they may fail in those cases where large structures are missing. On the contrary, our proposed data-driven completion framework provides a benefited solution for object completion in such extreme cases.

Specifically, using the pre-trained network on ShapeNet, real street images and incomplete object models are fed into the network to generate a complete model. Then, utilised Iterative Closest Point (ICP) registration method [26] provided in Point Cloud Library (PCL), the generated point clouds are aligned with the initial point clouds, followed by merging and normalizing to form complete models.

**Figure 12:**
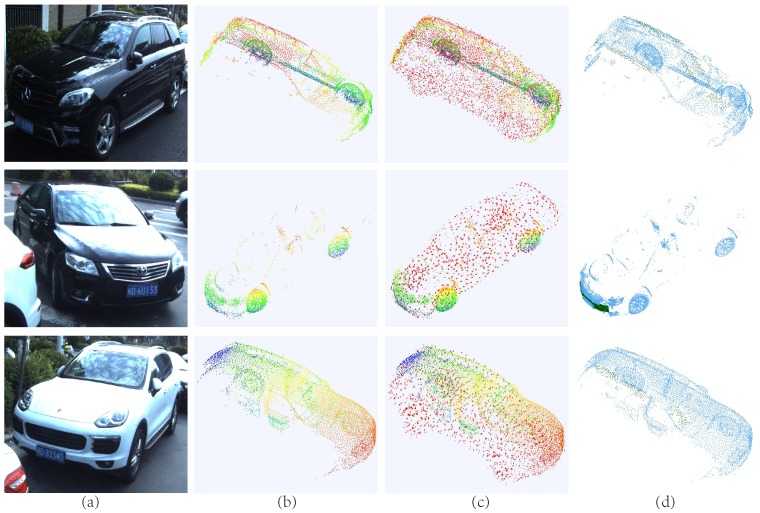
Results of the MLS objects completion. (**a**) Street images. (**b**) Original MLS point clouds (Missing more than a half). (**c**) The completion results of PCCNet. (**d**) The completion results of [8].

**Figure 13:**
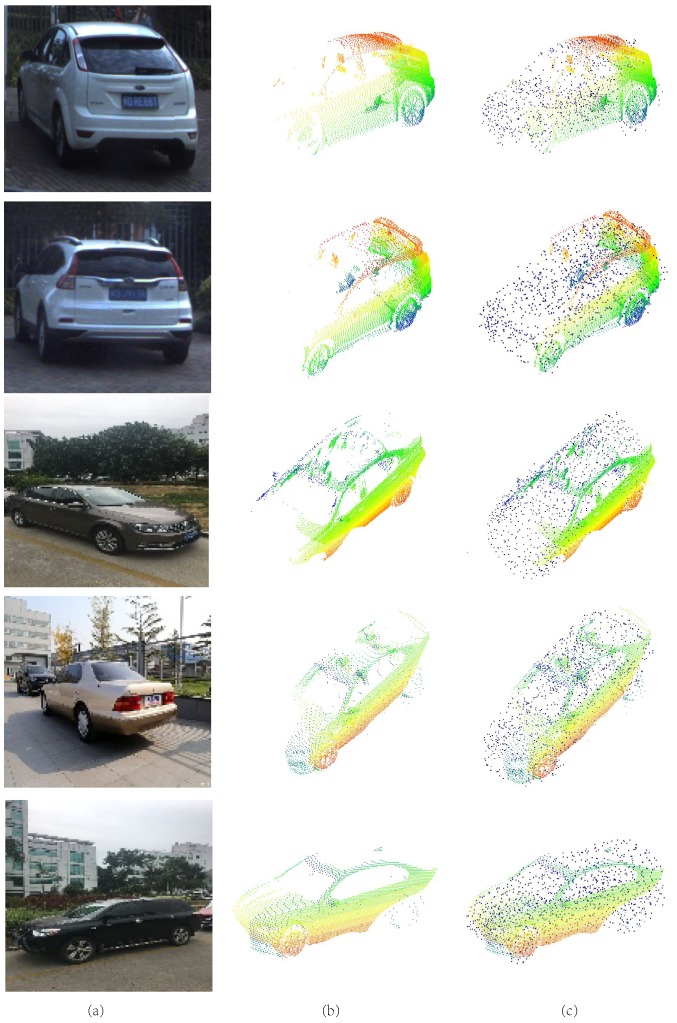
More results of MLS data. (**a**) Street images. (**b**) Original MLS point clouds. (**c**) The completion results of PCCNet.

The experiment results are shown in Figure 12 and Figure 13. Three different kinds of cars have different qualities in MLS point clouds. Among them, the white Porsche has relatively dense and intact scanning structures in the front, but it lacks 3D structures at the back. The Toyota in the middle row is the most incomplete, missing more than three quarters of the entire model. Figure 12a,b are the original street images and incomplete scanning models, forming the input pairs. Figure 12c displays the results of PCCNet, and it can be seen that no matter how large the missing parts are, our method can produce the entire models, which are almost identical to the actual 3D structures. As for the traditional completion methods [8], which represents state-of-the-art MLS completion standard. As shown in Figure 12d, under the same conditions, the method [8] fails to complete the large holes or fill in wrong places.

Limited by the categories of ShapeNet models, in this paper, we only train and test on the cars, as shown in Figure 13. It can be seen that our method, along the data-driven way, can produce complete models for the largely incomplete shapes, where previous feature-based methods may probably fail. As for other categories, we have confidence that our method is also suitable for them.

## 4. Conclusions

We designed a novel generative network that is more suitable for point cloud objects completion. Instructed by 2D street images, our method can infer the 3D missing structures based on 2D information. Additionally, by adding the projection loss of the generated point clouds, the network achieves higher accuracy. Our network is the first image-guided deep-learning-based method for the point cloud objects completion task. Experiments show that our method performs well for 3D reconstruction and 3D objects completion under the real environment set. However, limited by the categories of ShapeNet, we only train and test on cars, but our method is also suitable for other categories, such as traffic lights, bus station, buildings, etc. The unified deep-learning architecture of combining 2D and 3D feature is a worth and promising issue in 3D modeling and processing, the proposed network has provided an efficient way to integrate 2D and 3D information to guide the point cloud completion. 

## Figures and Tables

**Table 1:** CD scores of PSGN and PCCNet.

Category	PSGN (CD)	PCCNet_WP	PCCNet_WF	PCCNet_P
Sofa	0.00220	0.00201	0.00195	0.00161
Airplane	0.00100	0.00084	0.00092	0.00071
Bench	0.00251	0.00233	0.00231	0.00195
Car	0.00128	0.00136	0.00127	0.00123
Chair	0.00238	0.00210	0.00191	0.00181

**Table 2:** IoU scores of OGN and PCCNet.

Category	OGN (IoU)	PCCNet_WP	PCCNet_WF	PCCNet_P
Sofa	0.11204	0.19014	0.19310	0.21018
Airplane	0.14727	0.34216	0.28621	0.43376
Bench	0.04608	0.25839	0.26517	0.27712
Car	0.44141	0.31326	0.31591	0.33721
Chair	0.13935	0.20318	0.24133	0.25320
